# Differential Indicators of Diabetes-Induced Oxidative Stress in New Zealand White Rabbits: Role of Dietary Vitamin E Supplementation

**DOI:** 10.1080/15604280214279

**Published:** 2002

**Authors:** Randall L. Davis, Christy L. Lavine, Melissa A. Arredondo, Patrick McMahon, Thomas E. Tenner, Jr.

**Affiliations:** 1 Department of Pharmacology Texas Tech University Health Sciences Center Lubbock Texas 79430 USA

## Abstract

Determination of reliable bioindicators of diabetes-induced oxidative stress and the role of dietary vitamin E supplementation were investigated. Blood (plasma) chemistries, lipid peroxidation (LPO), and antioxidant enzyme activities were measured over 12 weeks in New Zealand White rabbits (control, diabetic, and diabetic + vitamin E). Cholesterol and triglyceride levels did not correlate with diabetic state. PlasmaLPOwas influenced by diabetes and positively correlated with glucose concentration only, not cholesterol or triglycerides. Liver glutathione peroxidase (GPX) activity negatively correlated with glucose and triglyceride levels. Plasma and erythrocyte GPX activities positively correlated with glucose, cholesterol, and triglyceride concentrations. Liver superoxide dismutase activity positively correlated with glucose and cholesterol concentration. Vitamin E reduced plasma LPO, but did not affect the diabetic state. Thus, plasmaLPOwas the most reliable indicator of diabetes-induced oxidative stress. Antioxidant enzyme activities and types of reactive oxygen species generated were tissue dependent. Diabetes-induced oxidative stress is diminished by vitamin E supplementation.


*Int. Jnl. Experimental Diab. Res.*, 3:185–192, 2002
Copyright © 2002 Taylor & Francis
1560-4284/02 $12.00 + .00
DOI: 10.1080/15604280290013892

### Differential Indicators of Diabetes-Induced Oxidative Stress in New Zealand White Rabbits: Role of Dietary Vitamin E Supplementation

**Randall L. Davis, Christy L. Lavine, Melissa A. Arredondo, Patrick McMahon, and Thomas E. Tenner, Jr.**

*Department of Pharmacology, Texas Tech University Health Sciences Center, Lubbock, Texas, USA*

**Determination of reliable bioindicators of diabetes-induced oxidative stress and the role of dietary vitamin E supplementation were investigated. Blood (plasma) chemistries, lipid peroxidation (LPO), and antioxidant enzyme activities were measured over 12 weeks in New Zealand White rabbits (control, diabetic, and diabetic + vitamin E). Cholesterol and triglyceride levels did not correlate with diabetic state. Plasma LPO was influenced by diabetes and positively correlated with glucose concentration only, not cholesterol or triglycerides. Liver glutathione peroxidase (GPX) activity negatively correlated with glucose and triglyceride levels. Plasma and erythrocyte GPX activities positively correlated with glucose, cholesterol, and triglyceride concentrations. Liver superoxide dismutase activity positively correlated with glucose and cholesterol concentration. Vitamin E reduced plasma LPO, but did not affect the diabetic state. Thus, plasma LPO was the most reliable indicator of diabetes-induced oxidative stress. Antioxidant enzyme activities and types of reactive oxygen species generated were tissue dependent. Diabetes-induced oxidative stress is diminished by vitamin E supplementation.**

**Keywords** Antioxidant; Oxidative Stress; Type 1 Diabetes; Vitamin E

Oxidative stress is a common consequence of diabetes mellitus [1–4]. Diabetic complications associated with oxidative stress include nephropathy, neuropathy, retinopathy, and vasculopathy [2, 5–9]. Approximately 80% of the death and disability associated with diabetes is due to cardiovascular disease secondary to atherosclerosis [10]. Consequently, there is a great deal of interest in understanding the role of oxidative stress in these diabetic complications. However, the literature remains unclear as to which biological measurements are the most reliable bioindicators of oxidative stress specifically associated with diabetes. It is also uncertain as to whether there may be tissue-specific responses to diabetes-induced oxidative stress.

Dietary supplementation with the antioxidant vitamin E has been suggested as a plausible means of controlling diabetic complications [11, 12]. Conversely, others have shown that dietary vitamin E supplementation is not useful in the prevention of oxidative stress associated with diabetes [13, 14].

To address these concerns, a type 1 diabetic rabbit model was used to investigate various assays/tissues in order to identify a reliable bioindicator of oxidative stress specifically associated with diabetes. Secondly, the effect of dietary vitamin E supplementation on diabetes-induced oxidative stress and the diabetic state was investigated.

### MATERIALS AND METHODS

### Animals

Following a 7-day acclimation period, juvenile, male, New Zealand White (NZW) rabbits (Charles River Laboratories, Wilmington, MA, USA) were injected with alloxan monohydrate (100 mg/kg body weight; Sigma, St. Louis, MO, USA) via the marginal ear vein in order to induce type 1 diabetes. This is a well-characterized model of type 1 diabetes. Rabbits were injected subcutaneously every 4 to 6 hours, for the first 24 hours, with a 40% glucose solution (2 g/kg

Received 4 March 2002; accepted 23 May 2002.
This material is based in part upon work supported by the Texas Advanced Research Program under grant no. 010674-039. The authors acknowledge Dr. Barbara C. Pence for her valuable consultation.
Address correspondence to Dr. T. E. Tenner, Jr., Department of Pharmacology, Texas Tech University Health Sciences Center, Lubbock, TX 79430, USA. E-mail: tom.tenner@ttuhsc.edu body weight) to prevent onset of acute hypoglycemia. Rabbits were randomly separated into 3 treatment groups: control ($n = 5$), diabetic ($n = 3$), and diabetic + vitamin E ($n = 4$). All rabbits were fed rabbit chow (Harlan Teklad, Madison, WI, USA) and water ad libitum. Two weeks after the alloxan injection, the diabetic + vitamin E group received a daily dietary supplement of vitamin E (500 mg/day; Bio-Serve, Frenchtown, NJ, USA), whereas the control and diabetic groups received placebo tablets (Bio-Serve). Palatability of the vitamin E and placebo supplements was excellent. Experiments were performed in accordance with the *Principles of Laboratory Animal Care* (NIH publication no. 85-23, revised 1995).

### Body Weight and Tissue Collection

Initial experimental samples/measurements were obtained prior to induction of diabetes or vitamin E supplementation. Subsequent samples/measurements were taken at 2, 4, 8, and 12 weeks. Following assessment of body weight, whole blood was collected via the central ear artery and transferred to a vacutainer tube (lavender cap with $K_3$EDTA; Becton Dickinson, Franklin Lakes, NJ, USA) and placed on ice. Plasma was collected by centrifugation ($400 \times g$, 15 minutes, 10°C) and stored at −80°C until needed for analysis. Erythrocytes were collected and washed 3 times with phosphate-buffered saline (PBS) (pH 7.4). Erythrocytes were then sonicated on ice for 20 seconds, centrifuged at $30{,}000 \times g$ for 30 minutes at 4°C, and then the supernatants were stored at −80°C.

At 12 weeks, whole blood was collected via cardiac puncture from anesthetized (ketamine 50 mg/kg + xylazine 10 mg/kg) rabbits. Rabbits were then euthanized with a single cardiac injection of Fatal Plus (concentrated pentobarbital, 360 mg/kg). Liver tissue was collected into 0.25 mol/l sucrose and stored immediately on ice. Subsequently, liver tissue was homogenized (Polytron, Brinkman Instruments, Westbury, NJ, USA) on ice for 20 seconds at setting = 6; then centrifuged at $30{,}000 \times g$ for 30 minutes at 4°C. The supernatant was collected and stored at −80°C.

### Blood Chemistries

Glucose, cholesterol, and triglyceride concentrations in plasma were determined using commercially available kits (Sigma).

### Lipid Peroxidative Damage

Lipid peroxidative (LPO) damage was quantitated by measuring thiobarbituric acid reactive substances (TBARS) in the plasma, using a modified method of Agil et al. [15]. The TBARS assay is a routinely utilized method for assessing LPO [16–18]. The primary goal of this study was to determine a reliable bioindicator of oxidative stress specifically associated with diabetes, not to quantitate the absolute amount of LPO. Briefly, for this method, 20 $\mu$l of plasma was combined with 4 ml of 0.08 N $H_2SO_4$ and 0.5 ml of 10% phosphotungstic acid (Sigma), and centrifuged at $400 \times g$ for 20 minutes at 25°C. The pellet was then resuspended in 2 ml 0.08 N $H_2SO_4$ and 0.5 ml of 10% phosphotungstic acid and centrifuged as before. The pellet was then mixed with 1 ml of thiobarbituric acid assay reagent (mixture of equal volumes of a 4.6 mol/l 2-thiobarbituric acid [Sigma] aqueous solution and glacial acetic acid) and 4 ml of dd$H_2O$ and placed in a boiling water bath for 60 minutes. Next, 5 ml of n-butanol was added and the tubes centrifuged as before for 30 minutes. Fluorescence of the butanol layer was measured at excitation and emission wavelengths of 515 nm and 553 nm, respectively, using a spectrofluorometer (model RF-1501, Shimadzu, Japan). Malondialdehyde (MDA; Sigma) was used to generate a standard curve and TBARS were expressed as MDA equivalents (nmol)/ml plasma.

### Glutathione Peroxidase Activity

Selenium-dependent glutathione peroxidase (GPX) activity in plasma, erythrocytes, and liver was measured using a modification of the method described by Paglia and Valentine [19]. Using 0.25 mmol/l $H_2O_2$ as substrate, the oxidation of NADPH was monitored over 180 seconds at 340 nm using a Shimadzu UV-visible spectrophotometer (Model 160, Kyoto, Japan). The assay mixture contained 2 mmol/l glutathione, 0.25 mmol/l NADPH, 1 U of glutathione reductase, 1.0 mmol/l sodium azide, 1.5 mol/l EDTA, and 50 mmol/l potassium phosphate buffer (pH 7.0). GPX activity is reported in mU/mg protein. Protein content of the plasma (or tissue supernatant) was determined using a commercially available protein quantitation assay (Bio-Rad Laboratory, Richmond, CA, USA).

### Superoxide Dismutase Activity

Total superoxide dismutase (SOD) activity was determined in plasma, erythrocytes, and liver according to a modified assay described by Pence and Naylor [20], in which the reduction of cytochrome *c* is measured over 2 minutes at 550 nm using a Shimadzu UV-visible spectrophotometer. Briefly, 50 $\mu$l of sample was combined with 940 $\mu$l of the assay mixture (0.023 mmol/l cytochrome *c* [from horse heart, Sigma] and 0.04 mmol/l xanthine [Sigma] in buffer solution [50 mmol/l $NaKPO_4$; 0.1 mmol/l EDTA, pH 7.0]). To initiate the reaction, 0.18 U (or amount needed to produce a change in absorbance/min = 0.020–0.030) of xanthine oxidase (grade III; Sigma) was added to cuvette. One unit of SOD activity was defined as the amount of SOD needed to inhibit the rate of cytochrome *c* reduction by 50% and was expressed as units per milligram protein. Protein concentration was determined as described above.

### Statistical Analysis

All data are expressed as the mean ± SEM. Analysis of variance was used to determine if the treatments were significantly ($P < 0.05$) different. The Newman-Keuls method was used to separate means when significant treatment effects were observed. Linear-regression analysis was used to determine correlations between blood chemistries and indicators of oxidative stress. All statistical analyses were done using GraphPad Prism 2.0 (GraphPad Software, San Diego, CA, USA).

### RESULTS

### Blood Chemistries

Body weight was not significantly different between treatment groups through the first 4 weeks of treatment. At 8 and 12 weeks, diabetic rabbits had significantly lower body weights than control rabbits, whereas body weights of diabetic + vitamin E rabbits were not significantly different from either control or diabetic rabbits (Figure 1).

By 2 weeks, alloxan-treated rabbits were indeed diabetic, as indicated by plasma glucose levels greater than 300 mg/dL (Figure 2*A*) and remained hyperglycemic throughout the 12 weeks of the experiment. Dietary vitamin E supplementation had no effect on plasma glucose levels, as glucose levels were not significantly different from those in diabetic rabbits (Figure 2*A*).

Although plasma cholesterol and triglyceride levels exhibited a similar profile to that of plasma glucose, treatment groups were not significantly different from controls (Figure 2*B*, 2*C*). Indeed, cholesterol and triglyceride data revealed large variability

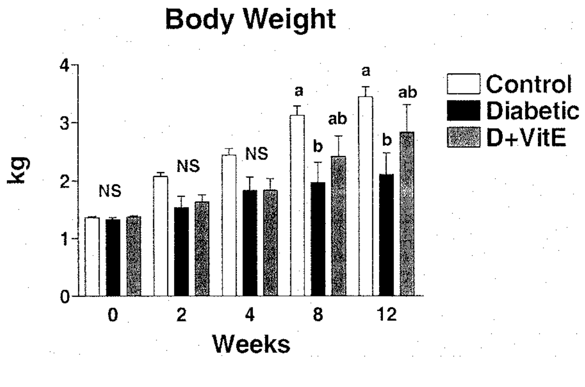

**FIGURE 1**
Body weights of control, diabetic, and diabetic + vitamin E–supplemented (500 mg/day) NZW rabbits over a 12-week period. Data represent mean ± SEM (n = 3–5). Data points with different, lowercase superscripts are significantly ($P < 0.05$) different and NS denotes nonsignificance.

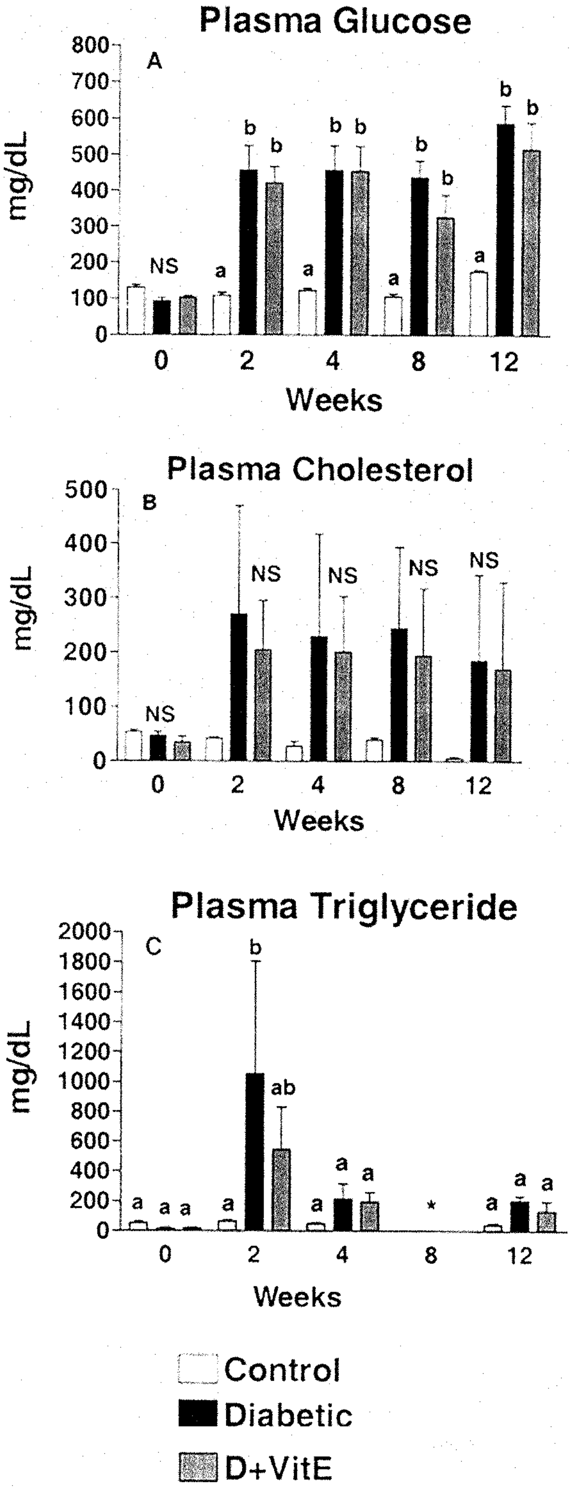

**FIGURE 2**
Plasma glucose, cholesterol, and triglyceride levels of control, diabetic, and diabetic + vitamin E–supplemented (500 mg/day) NZW rabbits over a 12-week period. Data represent mean ± SEM (n = 3–5). Data points with different, lower case superscripts are significantly ($P < 0.05$) different, NS denotes nonsignificance, and * indicates missing data.

among rabbits, resulting in inconsistent data. One exception was at 2 weeks, when plasma triglycerides were significantly elevated in the diabetic group (Figure 2*C*).

### Nonenzymatic Indicators of Oxidative Stress

Oxidative stress, as indicated by lipid peroxidation (TBARS) in the plasma, was increased 2-fold at 12 weeks in the diabetic group. The level of TBARSs in the vitamin E–supplemented group was significantly less than the diabetic group and not different from the control group (Figure 3*A*).

Linear-regression analysis demonstrated a significant positive correlation between plasma glucose concentration and levels of plasma TBARS at 12 weeks (Figure 3*B*). However, neither plasma cholesterol nor plasma triglyceride levels were correlated with plasma TBARS (data not shown).

### Enzymatic Indicators of Oxidative Stress

GPX activity was significantly decreased at 12 weeks in the liver of diabetic rabbits as compared to control rabbits, whereas liver GPX activity in vitamin E–supplemented rabbits was not significantly different from either of the other 2 treatment groups (Figure 4*A*). Linear-regression analysis indicated a significant, negative correlation between plasma glucose concentration and liver GPX activity at 12 weeks (Figure 4*B*). Plasma triglyceride concentration was also negatively correlated ($r^2 = .5995$, $P = .0086$) with liver GPX activity at 12 weeks, whereas plasma cholesterol levels were not correlated with liver GPX activity (data not shown).

GPX activity in plasma and erythrocytes was seemingly increased in the diabetic and vitamin E–supplemented rabbits at

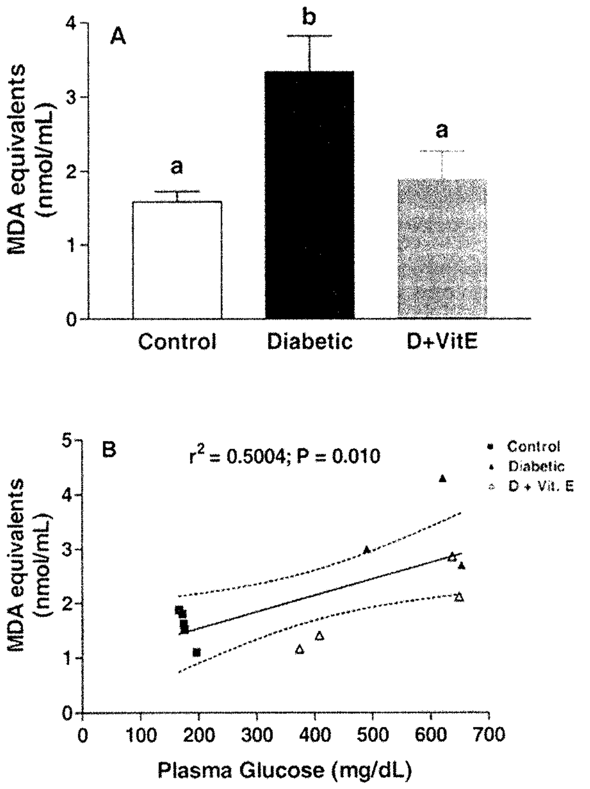

**FIGURE 3**

(*A*) Plasma lipid peroxidation (as determined by the TBARS assay) in control, diabetic, and diabetic + vitamin E–supplemented (500 mg/day) NZW rabbits following a 12-week treatment period. (*B*) Correlation between plasma lipid peroxidation and plasma glucose concentration after 12 weeks of treatment, as determined by linear regression analysis. Data represent mean ± SEM (n = 3–5). Data points with different, lowercase superscripts are significantly ($P < 0.05$) different.

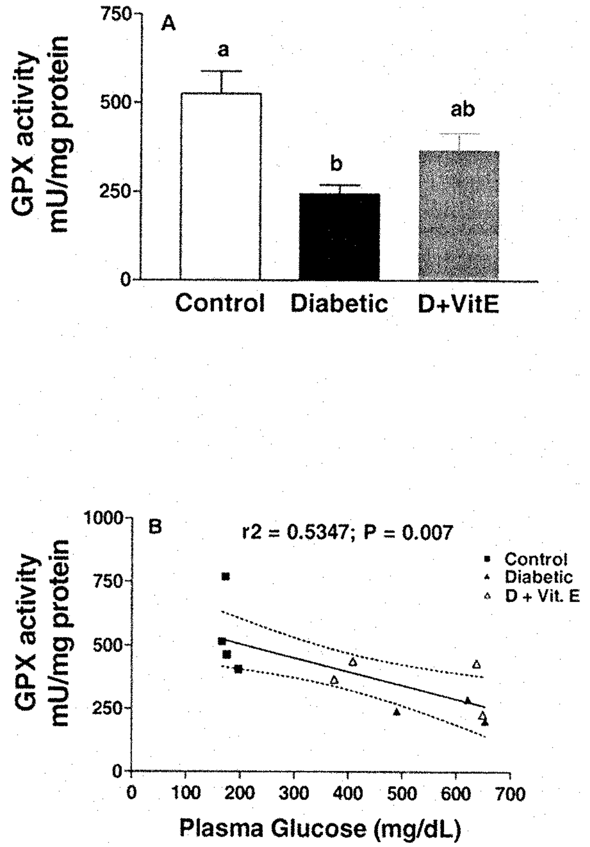

**FIGURE 4**

(*A*) Liver glutathione peroxidase (GPX) activity in control, diabetic, and diabetic + vitamin E–supplemented (500 mg/day) NZW rabbits following a 12-week treatment period. (*B*) Correlation between liver GPX activity and plasma glucose concentration after 12 weeks of treatment, as determined by linear-regression analysis. Data represent mean ± SEM (n = 3–4). Data points with different, lowercase superscripts are significantly ($P < 0.05$) different.

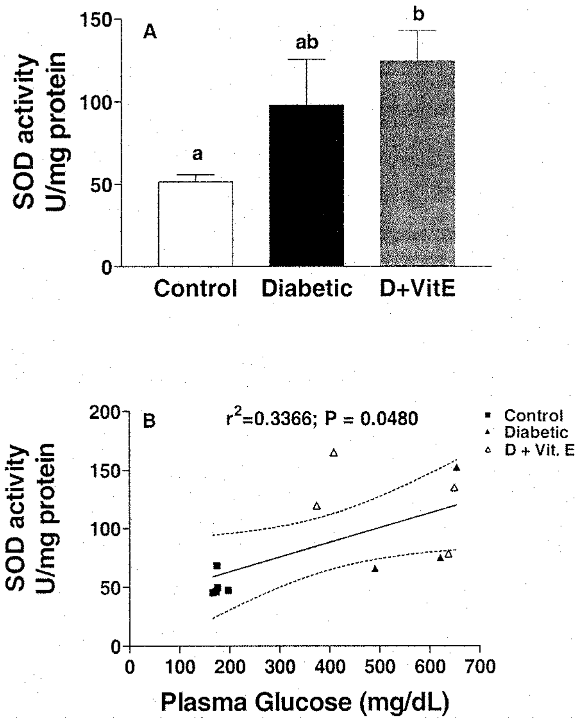

**FIGURE 5**

(*A*) Liver superoxide dismutase (SOD) activity in control, diabetic, and diabetic + vitamin E–supplemented (500 mg/day) NZW rabbits following a 12-week treatment period. (*B*) Correlation between liver SOD activity and plasma glucose concentration after 12 weeks of treatment, as determined by linear-regression analysis. Data represent mean ± SEM (n = 3–5). Data points with different, lowercase superscripts are significantly ($P < 0.05$) different.

12 weeks, however, not to the level of significance (data not shown). Liver SOD activity was increased approximately 2.5-fold at 12 weeks in the diabetic group receiving supplemental vitamin E, compared to control rabbits (Figure 5*A*). However, liver SOD activity in the diabetic rabbits was not significantly different from the other 2 groups. Linear-regression analysis demonstrated a significant, positive correlation ($r^2 = .3656$, $P = .0373$; Figure 5*B*) between plasma glucose concentration and liver SOD activity at 12 weeks. Plasma cholesterol and triglyceride were not significantly correlated with liver SOD activity. SOD activity in the plasma and erythrocytes was similar among all 3 treatment groups at 12 weeks and not correlated with any of the blood chemistries measured (data not shown).

### DISCUSSION

Oxidative stress is a well-documented consequence of diabetes mellitus [6, 7, 21]. The purpose of the present study was to evaluate which method/tissue would efficiently and reliably detect oxidative stress specifically associated with diabetes. Secondly, the ability of dietary vitamin E supplementation to reduce diabetes-induced oxidative stress and correct the diabetic state was examined. There are several methods commonly used to detect oxidative stress, including antioxidant enzyme status [22], nonenzymatic antioxidant status [3], LPO [23], DNA adduct formation [24], and isoprostane levels [25]. Typical tissues assayed for oxidative stress include plasma, erythrocytes, pancreas, kidney, and liver [20, 26, 27]. Although DNA adduct formation and isoprosanes are generally considered to be more specific, quantitative measures of oxidative stress, they are very expensive and relatively laborious. Therefore, we selected assays that are routinely used, including LPO and antioxidant enzyme activity, due to their ease of performance, repeatability, and cost-effectiveness [16, 28, 29].

A single injection of alloxan was sufficient to induce diabetes, as indicated by plasma glucose concentrations in the 400- to 600-mg/dL range by 2 weeks after injection (Figure 2*A*). Neither plasma cholesterol nor triglyceride concentrations were consistently elevated by diabetes. The apparent partial recovery of body weight in diabetic rabbits supplemented with vitamin E was not the result of a vitamin E–induced decrease in diabetic severity as defined by blood glucose. Vitamin E had no significant effect on diabetic blood glucose levels.

LPO in the plasma, as determined by the TBARS assay, was the most reliable indicator of diabetes-induced oxidative stress. This appeared to be the case, as plasma LPO was the only measure of oxidative stress that was positively and significantly correlated with plasma glucose concentration, but not plasma cholesterol or triglyceride concentrations, which were not consistently or significantly altered by the diabetic state.

The decrease in liver GPX activity at 12 weeks in diabetic rabbits may be indicative of an exhaustion of GPX as a consequence of increased oxidative stress. Similar decreases in liver GPX activity have been found in streptozotocin (STZ)-induced diabetic pregnant rats [30] and alloxan-induced diabetic rats [31]. Conversely, others have documented increased liver GPX activity associated with diabetes [32]. Liver GPX activity was negatively correlated with plasma glucose and triglyceride concentration but not plasma cholesterol. In contrast, GPX activity in the plasma and erythrocytes was not significantly affected by the diabetic state.

Decreased pancreatic GPX activity, with concurrent normal erythrocyte and plasma GPX activity, has been documented in prediabetic diabetic-prone BB rats [20]. These data further indicate that the response of GPX to oxidative stress appears to be tissue dependent, and, with respect to the liver, the literature suggests GPX activity is quite variable in response to oxidative stress [30–32]. Based upon the data presented here, as well as the literature, GPX activity does not appear to be a reliable indicator of diabetes-associated oxidative stress.

SOD activity was quite variable in all the tissues assessed, and, although correlated with the hyperglycemic state, the magnitude of the response was small and not significant, therefore, preempting SOD activity from reliably reflecting diabetic-induced oxidative state. Recently, Ruiz and colleagues [33] found that patients with type 1 diabetes had increased oxidative stress, as indicated by increased LPO; however, SOD activity was unaffected. In contrast, pregnant women with type 1 diabetes had decreased plasma SOD activity with a concurrent elevation in plasma LPO [34]. Wohaieb and Godin [35] suggest that the variable alterations in antioxidant enzyme activities in various tissues may reflect a compensatory response in those tissues that typically possess low levels of a particular enzyme and an inhibitory or exhaustive effect in tissues with increased tissue oxidant activity. Given that the substrates for GPX and SOD are different (hydrogen peroxide and superoxide, respectively), the differential response of these 2 enzymes to diabetes-induced oxidative stress may reflect differences in reactive oxygen species generated.

Evaluation of the various indicators of oxidative stress measured in the present study suggests that, in this rabbit model of type 1 diabetes, plasma LPO is a more reliable indicator of diabetes-induced oxidative stress than either GPX or SOD activity.

Vannucchi and colleagues [12] recently found in STZ-induced diabetic rats that vitamin E deficiency resulted in elevated LPO (as indicated by the TBARS assay), whereas vitamin E supplementation protected from LPO and returned glucose levels to normal. Similarly, vitamin E supplementation (100 IU/day) in patients with type 1 diabetes resulted in a 30% decrease in LPO as determined by the TBARS assay [36]. These investigators did not report blood glucose data following vitamin E supplementation; however, they did indicate insulin use by the patients was unaffected [36]. Data from the present study demonstrate that dietary vitamin E supplementation (500 IU/day) was sufficient to reduce diabetes-induced oxidative stress, but not plasma glucose concentration.

Control of diabetes-induced oxidative stress could theoretically be used to reduce the severity of diabetic complications. The antioxidant vitamin E has received much attention for its potential role in controlling oxidative stress [11, 12, 37]. With respect to dietary vitamin E and diabetes-induced oxidative stress, there is still much to be learned. Palmer and colleagues [37] found that vitamin E supplementation in STZ-induced diabetic rats decreased oxidative stress, as indicated by reduced levels of 8-epi prostaglandin F2 alpha in the plasma. Relatively high doses of vitamin E (900 mg/day for 4 months) have been found to reduce oxidative stress in diabetic patients, as determined by an indirect measurement of serum oxygen generation using a ferricytochrome *c* reduction–based assay, in the presence and absence of SOD [38]. Likewise, Astley and colleagues [27] measured DNA single-strand breaks in peripheral lymphocytes and susceptibility to hydrogen peroxide–induced stress (comet assay) as an indicator of oxidative stress, and found that diabetic patients were protected by vitamin E supplementation (400 IU/day for 8 weeks). Although there are studies that question the protective effects of vitamin E in the diabetic [39], the current study and those referred to above would indicate that an appropriate dose of vitamin E can modify diabetes-induced oxidative stress.

In conclusion, we have demonstrated in a rabbit type 1 diabetes model that plasma LPO is a reliable indicator of diabetes-induced oxidative stress, as it was positively correlated with plasma glucose concentration, but not with the variable plasma cholesterol or triglyceride concentrations. Conversely, the antioxidant enzyme (GPX and SOD) activities tested were less reliable bioindicators of oxidative stress specifically associated with diabetes. Dietary vitamin E supplementation significantly reduced diabetes-induced oxidative stress (as indicated by a decrease in plasma LPO), but had no effect on the hyperglycemic state. These findings support the role of dietary antioxidant supplementation in the prevention of diabetes-induced oxidative stress, and suggest a potential therapeutic benefit for the reduction of diabetic complications associated with oxidative stress.

### REFERENCES

[1] Dandona, P., Thusu, K., Cook, S., Snyder, B., Makowski, J., Armstrong, D., and Nicotera, T. (1996) Oxidative damage to DNA in diabetes mellitus. *Lancet,* **347,** 444–445.

[2] Giugliano, D., Ceriello, A., and Paolisso, G. (1996) Oxidative stress and diabetic vascular complications. *Diabetes Care,* **19,** 257–263.

[3] Feillet-Coudray, C., Rock, E., Coudray, C., Grzelkowska, K., Azais-Braesco, V., Dardevet, D., and Mazur, A. (1999) Lipid peroxidation and antioxidant status in experimental diabetes. *Clin. Chim. Acta,* **284,** 31–43.

[4] Rosen, P., Nawroth, P. P., King, G., Moller, W., Tritschler, H. J., and Packer, L. (2001) The role of oxidative stress in the onset and progression of diabetes and its complications: A summary of a Congress Series sponsored by UNESCO-MCBN, the American Diabetes Association and German Diabetes Society. *Diabetes Metab. Res. Rev.,* **17,** 189–212.

[5] Van Dam, P. S., Van Asbeck, B. S., Erkelens, D. W., Marx, J. M., Gispen, W. H., and Bravenboer, B. (1995) The role of oxidative stress in neuropathy and other diabetic complications. *Diabetes Metab. Res. Rev.,* **11,** 181–192.

[6] Sharpe, P. C., Liu, W. H., Yue, K. K., McMaster, D., Catherwood, M. A., McGinty, A. M., and Trimble, E. R. (1998) Glucose-induced oxidative stress in vascular contractile cells: Comparison of aortic smooth muscle cells and retinal pericytes. *Diabetes,* **47,** 801–809.

[7] Greene, D. A., Stevens, M. J., Obrosova, I., and Feldman, E. L. (1999) Glucose-induced oxidative stress and programmed cell death in diabetic neuropathy. *Eur. J. Pharmacol.,* **375,** 217–223.

[8] Ha, H., and Kim, K. H. (1999) Pathogenesis of diabetic nephropathy: The role of oxidative stress and protein kinase C. *Diabetes Res. Clin. Pract.,* **45,** 147–151.

[9] Hinokio, Y., Suzuki, S., Hirai, M., Chiba, M., Hirai, A., and Toyota, T. (1999) Oxidative DNA damage in diabetes mellitus: Its association with diabetic complications. *Diabetologia,* **42,** 995–998.

[10] Kannel, W. B., and McGee, D. L. (1979) Diabetes and cardiovascular disease: The Framington study. *JAMA,* **241,** 2035–3038.

[11] Rosen, P., Du, X., and Tschope, D. (1998) Role of oxygen derived radicals for vascular dysfunction in the diabetic heart: Prevention by alpha-tocopherol? *Mol. Cell Biochem.,* **188,** 103–111.

[12] Vannucchi, H., Araujo, W. F., Bernardes, M. M., and Jordao, A. A. (1999) Effect of different vitamin E levels on lipid peroxidation in streptozotocin-diabetic rats. *Int. J. Vitam. Nutr. Res.,* **69,** 250–254.

[13] Mayer-Davis, E. J., Bell, R. A., Reboussin, B. A., Rushing, J., Marshall, J. A., and Hamman, R. F. (1998) Antioxidant nutrient intake and diabetic retinopathy: The San Luis Valley diabetes study. *Ophthalmology,* **105,** 2264–2270.

[14] Gazis, A., White, D. J., Page, S. R., and Cockcroft, J. R. (1999) Effect of oral vitamin E (alpha-tocopherol) supplementation on vascular endothelial function in type 2 diabetes mellitus. *Diabetes Med.,* **16,** 304–311.

[15] Agil, A., Fuller, C. J., and Jialal, I. (1995) Susceptibility of plasma to ferrous ion/hydrogen peroxide-mediated oxidation: Demonstration of a possible Fenton reaction. *Clin. Chem.,* **41,** 220–225.

[16] D'Angelo, S., Ingrosso, D., Perfetto, B., Baroni, A., Zappia, M., Lobianco, L. L., Tufano, M. A., and Galletti, P. (2001) UVA irradiation induces L-isoaspartyl formation in melanoma cell proteins. *Free Radic. Biol. Med.,* **31,** 1–9.

[17] Kapsokefalou, M., and Miller, D. D. (2001) Iron loading and large doses of intravenous ascorbic acid promote lipid peroxidation in whole serum in guinea pigs. *Br. J. Nutr.,* **85,** 681–687.

[18] Santos, M. S., Santos, D. L., Palmeira, C. M., Seica, R., Moreno, A. J., and Oliveira, C. R. (2001) Brain and liver mitochondria isolated from diabetic Goto-Kakizaki rats show different susceptibility to induced oxidative stress. *Diabetes Metab. Res. Rev.,* **17,** 223–230.

[19] Paglia, D. E., and Valentine, W. N. (1967) Studies on the quantitative and qualitative characterization of erythrocyte glutathione peroxidase. *J. Lab. Clin. Med.,* **70,** 158–169.

[20] Pence, B. C., and Naylor, M. F. (1990) Effects of a single-dose ultraviolet radiation on skin superoxide dismutase, catalase, and xanthine oxidase in hairless mice. *J. Invest. Dermatol.,* **95,** 213–216.

[21] Ceriello, A. (1999) Hyperglycemia: The bridge between non-enzymatic glycation and oxidative stress in the pathogenesis of diabetic complications. *Diabetes Nutr. Metab.,* **12,** 42–46.

[22] L'Abbe, M. R., and Trick, K. D. (1994) Changes in pancreatic glutathione peroxidase and superoxide dismutase activities in the prediabetic diabetic-prone BB rat. *Proc. Soc. Exp. Biol. Med.,* **207,** 206–212.

[23] Rodriguez-Martinez, M. A., Alonso, M. J., Redondo, J., Salaices, M., and Marin, J. (1998) Role of lipid peroxidation and the glutathione-dependent antioxidant system in the impairment of endothelium-dependent relaxations with age. *Br. J. Pharmacol.,* **123,** 113–121.

[24] Suzuki, S., Hinokio, Y., Komatu, K., Ohtomo, M., Onoda, M., Hirai, S., Hirai, M., Hirai, A., Chiba, M., Kasuga, S., Akai, H., and Toyota, T. (1999) Oxidative damage to mitochondrial DNA and its relationship to diabetic complications. *Diabetes Res. Clin. Pract.,* **45,** 161–168.

[25] Liu, T., Stem, A., Roberts, L. S., and Morrow, J. D. (1999) The isoprostanes: Novel prostaglandin-like products of the free radical-catalyzed peroxidation of arachidonic acid. *J. Biomed. Sci.,* **6,** 226–235.

[26] Torres, M. D., Canal, J. R., and Perez, C. (1999) Oxidative stress in normal and diabetic rats. *Physiol. Res.,* **48,** 203–208.

[27] Astley, S., Langrish-Smith, A., Southon, S., and Sampson, M. (1999) Vitamin E supplementation and oxidative damage to DNA and plasma LDL in type 1 diabetes. *Diabetes Care,* **22,** 1626–1631.

[28] Aydin, A., Orhan, H., Sayal, A., Ozata, M., Sahin, G., and Isimer, A. (2001) Oxidative stress and nitric oxide related parameters in type II diabetes mellitus: Effects of glycemic control. *Clin. Biochem.,* **34,** 65–70.

[29] Kesavulu, M. M., Rao, B. K., Giri, R., Vijaya, J., Subramanyam, G., and Apparao, C. (2001) Lipid peroxidation and antioxidant enzyme status in type 2 diabetics with coronary heart disease. *Diabetes Res. Clin. Pract.,* **53,** 33–39.

[30] Kinalski, M., Telejko, B., Zarzkycki, W., Gorski, J., and Kinalska, I. (1998) The effect of vitamin E on antioxidant tissue activity in pregnant rats with streptozotocin-induced diabetes. *Przegl. Lek.,* **55,** 320–324.

[31] Saxena, A. K., Srivastava, P., Kale, R. K., and Baquer, N. Z. (1993) Impaired antioxidant status in diabetic rat liver: Effect of vanadate. *Biochem. Pharmacol.,* **45,** 539–542.

[32] Kakkar, R., Mantha, S. V., Radhi, J., Prasad, K., and Kalra, J. (1998) Increased oxidative stress in rat liver and pancreas during progression of streptozotocin-induced diabetes. *Clin. Sci. (Colch),* **94,** 623–632.

[33] Ruiz, C., Algeria, A., Barbera, R., Farre, R., and Lagarda, M. J. (1999) Lipid peroxidation and antioxidant enzyme activities in patients with type 1 diabetes mellitus. *Scand. J. Clin. Lab. Invest.,* **59,** 99–105.

[34] Kinalski, M., Telejko, B., Kowalska, I., Urban, J., and Kinalska, I. (1999) The evaluation of lipid peroxidation products and antioxidative enzymes activity in cord blood and placental homogenates of pregnant diabetic women. *Ginekol. Pol.,* **70,** 57–61.

[35] Wohaieb, S. A., and Godin, D. V. (1987) Alterations in free radical tissue-defense mechanisms in streptozotocin-induced diabetes in rat: Effects of insulin treatment. *Diabetes,* **36,** 1014–1018.

[36] Jain, S. K., Krueger, K. S., McVie, R., Jaramillo, J. J., Palmer, M., and Smith, T. (1998) Relationship of blood thromboxane-B2 (TxB2) with lipid peroxides and effect of vitamin E and placebo supplementation on TxB2 and lipid peroxide levels in type 1 diabetic patients. *Diabetes Care,* **21,** 1511–1516.

[37] Palmer, A. M., Thomas, C. R., Gopaul, N., Dhir, S., Anggard, E. E., Poston, L., and Tribe, R. M. (1998) Dietary antioxidant supplementation reduces lipid peroxidation but impairs vascular function in small mesenteric arteries of the streptozotocin-diabetic rat. *Diabetologia,* **41,** 148–156.

[38] Paolisso, G., D'Amore, A., Giugliano, D., Ceriello, A., Varricchio, M., and D'Onofrio, D. (1993) Pharmacologic doses of vitamin E improve insulin action in healthy subjects and noninsulin-dependent diabetic patients. *Am. J. Clin. Nutr.,* **57,** 650–656.

[39] Trachtman, H., Futterweit, S., Maesaka, J., Ma, C., Valderrama, E., Fuchs, A., Tarectecan, A. A., Rao, P. S., Sturman, J. A., and Boles, T. H. (1995) Taurine ameliorates chronic streptozotocin-induced diabetic nephropathy in rats. *Am. J. Physiol.,* **269,** F429–F438.

